# Quenching of zonal winds in Jupiter’s interior

**DOI:** 10.1073/pnas.2402859121

**Published:** 2024-06-10

**Authors:** Ulrich R. Christensen, Paula N. Wulff

**Affiliations:** ^a^Max Planck Institute for Solar System Research, 37077 Göttingen, Germany

**Keywords:** Jupiter, interior dynamics, magnetohydrodynamics

## Abstract

Gravity and magnetic field data obtained by the Juno mission show that Jupiter’s strong zonal winds extend a few thousand kilometers into the interior, but are quenched above the level where the electrical conductivity becomes significant. Here, we extend a simple linearized model [Christensen et al., *Astrophys. J.* **890**, 61 (2020)] that explains the braking of the jets by the combination of stable stratification and electromagnetic effects. We show that in the inviscid limit, the process is essentially governed by a single parameter, which we call the MAC-number (for the forces acting on the flow—Magnetic, Archimedian, and Coriolis). The predictions for the drop-off of the zonal winds agree well with results from 3D-convection models. We run calculations that take the full range of density and electrical conductivity variations in the top 5,600 km of Jupiter into account. In order to satisfy constraints on the power driving the jets and on their effect on Jupiter’s magnetic field, the top of the stable layer and the region where the jet velocity drops sharply must be near 2,000 km depth. The dissipation associated with quenching of the jets increases toward the poles, which can partly explain why the jets near ±20^°^ are faster than those at higher latitude.

## Significance

Strong east-west directed jets dominate the circulation on Jupiter. Gravity and magnetic data show that they extend deep into the planet, but are quenched above the point where the rising electrical conductivity reaches the value of sea water. The lack of solid boundaries in gas planets requires a special mechanism for braking the jets. A simple model including a layer that is stable against radial motion explains all observational constraints when the jets are quenched near 2,000 km depth. We identify a single parameter, describing the influence of gravitational, rotational, and electromagnetic forces, which controls the results. These agree well with results from a full 3D convection model. Our model could also be applied to Saturn or to gaseous exoplanets.

Tracking of Jupiter’s clouds has revealed that its banded surface pattern is associated with multiple vigorous jet streams, alternating with latitude between eastward and westward flow. Their velocity exceeds 100 m/s at low latitudes and is a few tens of m/s at higher latitudes. Measurements of Jupiter’s gravity field by NASA’s Juno mission suggest that the winds extend downward by some thousand kilometers (1). This interpretation did not go unchallenged. The nonuniqueness in the inversion of gravity anomalies allows, in principle, for an origin associated with dynamical processes in the interior that are unrelated to the surface winds (2). However, more recent analyses of the gravity signal strengthened the case for their relation to a deep continuation of the surface winds (3). They also support the assumption of the flow aligning with the planet’s rotation axis rather than continuing in radial direction (4), as is expected on dynamical grounds.

The electrical conductivity in Jupiter is negligible near the surface but rises sharply with depth in the top several thousand kilometers. If high wind velocity persisted down to where the electrical conductivity becomes significant, the secular variation of Jupiter’s magnetic field would be larger than observed (5) and the magnetic field morphology should be distorted by the wind shear (6). The observed secular variation has been modeled as advection by zonal flow with ≲1 cm/s (7), assumed to be unrelated to the surface winds. Strong effects associated with the surface winds would occur when a local magnetic Reynolds number (Rm) associated with their deep extension gets larger than order one at some depth (6). For a widely used conductivity model (8) Rm would exceed one near 2,500 km if the wind velocity had not started to drop above this depth. The combined constraints from the gravity and magnetic field data suggest that the jet velocity remains rather constant down to about 2,000 km but drops sharply below (9).

The jets are driven by an eddy momentum flux from small-scale convection to large-scale zonal flow (10). Numerical models of convection in a rotating spherical shell (11, 12) produce strong zonal winds when the boundaries are stress-free. Friction at a rigid lower boundary, which may serve as a proxy for magnetic forces impeding the flow, inhibits winds at higher latitudes, inside the so-called tangent cylinder (a cylinder aligned with the polar axis, touching the inner boundary at the equator) (13). Models which combine magnetic field generation in a deep conducting region with convective flow in a poorly conducting outer shell (14, 15) indeed show strong zonal surface winds at low latitudes, but none at high latitudes.

An additional ingredient seems to be required to allow for strong winds inside the tangent cylinder. Stable stratification in the right depth range is a promising candidate. Demixing of hydrogen and helium in the gas planets is a possible cause for stable stratification (16), but it would only occur at depths where the jets have vanished. Stratification at shallower depth has been proposed in order to simultaneously match the gravity moments and the atmospheric element abundance (17–19), but the actual depth range and its physical origin remain uncertain. The existence of a stable radiative zone, originally suggested by Guillot and Gautier (20), has recently been supported by the finding of low abundances of alkali metals (a major source of opacity) in Jupiter’s deep atmosphere (21). According to ref. 20, the radiative zone would exist in the temperature range 1,200 to 3,000 K, corresponding to about 500 to 2,000 km depth, which may be too shallow for playing a role in the truncation of the winds. We base our analysis on the hypothesis of the existence of stable stratification in the depth range where the wind velocity drops off, even though independent evidence for it remains scanty.

The dynamical role of a stable layer, in combination with electromagnetic forces, for the quenching of winds has been demonstrated in simple axisymmetric models with an imposed dipolar field where the winds are driven by an ad hoc force (22). A weak meridional circulation that is associated with the east-west winds (23), similar to Ferrel cells in the Earth’s atmosphere, perturbs the density stratification in the stable layer, turning it baroclinic. In accord with a thermal wind balance the zonal velocity decreases with depth. These models also showed that the influence of electromagnetic forces, acting on the meridional flow, makes the drop-off of the zonal winds very sharp near the top of the stable region.

Some recent 3D models that cover the full range from the deep dynamo region to a nearly insulating outer convection zone included a stably stratified layer sandwiched between the two (24, 25). They confirmed that such a layer allows for strong surface winds at higher latitudes which are quenched inside the stable region. These simulations are computationally very demanding. Simpler 3D models that involve only an upper convective region and a stable layer, with a magnetic field of strength B imposed, are more amenable for a systematic study, with the focus on the location and sharpness of the drop-off of the winds. Purely hydrodynamic simulations (26) showed that the penetration distance decreases with the degree of stability. Simulations with exponentially varying conductivity σ demonstrated that, for a fixed degree of stability, the combination $\sigma B^{2}$ controls how steeply the zonal wind velocity drops in the stable layer (27).

All 3D convection simulations suffer from the fact that they are overly viscous and some control parameters differ vastly from their planetary values. In ref. 22, we developed linearized 2D Cartesian box models of jet flow in a conducting stable layer. The simplifications allowed us to reach Jupiter-like parameters. Here, we build on this concept and extend it to the inviscid limit. We isolate a single parameter combination that controls the decay of zonal winds in a stably stratified conducting region. While the original model was restricted to polar latitudes, where gravity, rotation, and dipolar magnetic field nearly align, we now extend it to arbitrary angles between the different vectors. We also run the 3D simulations of (27) at lower viscosity in order to corroborate some of the scaling laws based on the simple model. Finally, we apply the Cartesian models to Jupiter, covering the full range of density and conductivity variations from the 1-bar level to 5,600 km depth.

## Linearized Cartesian Models in the Inviscid Limit

### Basic Concept and Equations.

Building on the concept introduced in ref. 22, we elaborate on a simple Cartesian model for the damping of zonal flow in a stably stratified region including the action of magnetohydrodynamic forces. Here, we first simplify it further by dropping viscosity and other terms in the equations that turned out to be insignificant. This reduces the problem to one that is governed by a single nondimensional control parameter.

We start out from the anelastic equations for magnetohydrodynamics (MHD) flow in a rotating system including buoyancy forces, the electromagnetic induction equation, and an advection-diffusion equation for codensity (e.g., ref. 28).

[1] $$\begin{array}{c} \partial u/\partial t+u\cdot \nabla u=-\nabla P-2\Omega \times u+F_{\nu }+\frac{J\times B}{\tilde{\rho }}+Cg, \end{array}$$

[2] $$\begin{array}{c} \partial B/\partial t=\nabla \times \left(u\times B\right)+\nabla \times \left(\frac{1}{\mu_{o}\sigma }\nabla \times B\right), \end{array}$$

[3] $$\begin{array}{c} \partial C/\partial t+u\cdot \nabla C=\frac{1}{\tilde{\rho }}\nabla \cdot \left(\tilde{\rho }\;\kappa \nabla C\right), \end{array}$$

[4] $$\begin{array}{c} \nabla \cdot \tilde{\rho }u=0\;\;,\;\;\nabla \cdot B=0. \end{array}$$

Here, u is the velocity vector, B magnetic field, $\tilde{\rho }$ background density, $J=\mu_{o}^{-1}\nabla \times B$ electrical current density, P an effective pressure, and C the codensity (28), i.e., the (negative) density anomaly of thermal or compositional origin normalized by $\tilde{\rho }$. Ω is the rotation frequency, $F_{\nu }$ viscous force (neglected below), g gravity, κ thermo-compositional diffusivity, and $\mu_{o}$ magnetic permeability. All are assumed to be constant, except for σ and $\tilde{\rho }$, which may vary with depth.

**Fig. 1.**
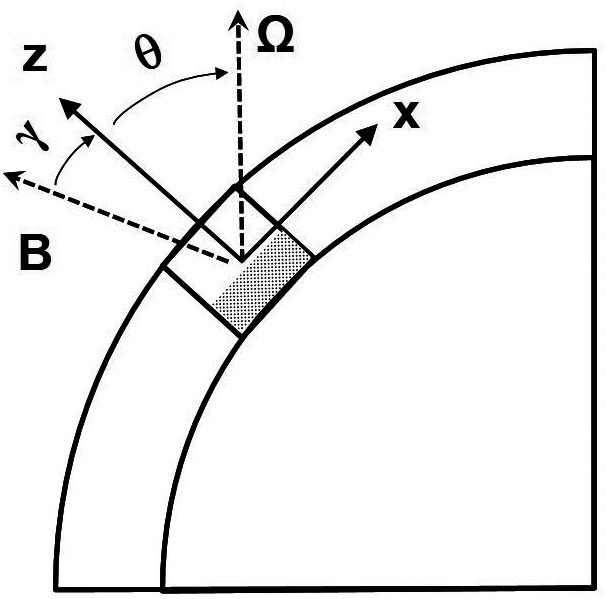
Schematic view for the setup of the linearized models. A Cartesian rectangle is cut out from a cross-section of the outer spherical shell region of a gas planet. Its lower part (shaded) is stably stratified. The z-coordinate is antiparallel to gravity g, x points north and y points west. In general, the orientation of the rotation vector Ω and the magnetic field vector B are at oblique angles, θ and γ, respectively, relative to gravity. All three vectors are assumed constant within the rectangle.

A torus in the outer layers of the planet is approximated by a rectangular Cartesian box (Fig. 1), where z is antiparallel to gravity and x corresponds to latitude. No variation in the perpendicular y-direction, representing longitude, is assumed. The background codensity is set to zero (neutral stability) in the upper part of the box at z>0, whereas a constant gradient in codensity is assumed in the stable lower region z<0. The Brunt-Väisälä frequency $N={\left(g\;dC/dz\right)}^{1/2}$ characterizes the degree of stability. We impose a uniform magnetic field B in the x-z-plane. We solve for the codensity perturbation c, assumed to be small compared to the background codensity C. We drop all nonlinear terms including momentum advection and assume a steady state. In addition to these simplifications already made in ref. 22, we drop the viscous term in Eq. **1** and neglect perturbations of the poloidal magnetic field, which is equivalent to setting $J_{y}=0$. Both had been found in ref. 22 to be negligible for planetary parameter values. Because of the linearity, we can expand all variables in Fourier series in the x-direction. For each horizontal wavenumber k we obtain a set of coupled ordinary differential equations.

While the cartoon in Fig. 1 shows a more general setting that we treat later, we start out by considering the special case where g, B, and Ω are parallel, like in ref. 22. Furthermore, we begin with the incompressible (Boussinesq) limit, i.e., $\tilde{\rho }=\rho_{o}$. We assume an exponential variation of electrical conductivity, $\sigma \left(z\right)=\sigma_{o}\;exp\left(-z/d_{\sigma }\right)$, with $\sigma_{o}$ the value at the upper boundary of the stable region at z = 0, and $d_{\sigma }$ the conductivity scale height. This, together with the other approximations and by scaling the variables in a suitable way, allows us to reduce the system of equations to a very compact set. We use the following dependent variables, $U=u_{y}$ for the zonal flow, the stream function ψ for the meridional circulation with $u=\nabla \times \psi e_{y}$, $b=B_{y}$ for the induced toroidal magnetic field, and the codensity perturbation c. Nondimensional variables, indicated by a tilde, are obtained by scaling length with $d_{\sigma }$ and by converting the dependent variables according to the following scheme (which differs from the scaling used in ref. 22):

[5] $$\begin{array}{c} U=\tilde{U}\;\frac{\kappa }{d_{\sigma }}\;,\;\;\;\;\;\;\psi =\tilde{\psi }\;\frac{\kappa \sigma_{o}B^{2}}{\rho_{o}\Omega }, \end{array}$$

[6] $$\begin{array}{c} b=\tilde{b}\;\kappa \sigma_{o}\mu_{o}B\;,\;\;\;\;\;\;c=\tilde{c}\;\frac{\sigma_{o}d_{\sigma }B^{2}N^{2}}{\rho_{o}g\Omega }. \end{array}$$

We expand U, ψ, and b in terms of sine functions, e.g. U=U(z)sin(kx), and c in cosine functions. For a given wavenumber k we obtain the following nondimensional equations, where we drop the tilde for simplicity and use shorthand $d_{z}$ and $d_{\mathrm{zz}}$ for the first and second derivative, respectively:

[7] $$\begin{array}{c} 2d_{z}\psi \;=\;-d_{z}b, \end{array}$$

[8] $$\begin{array}{c} d_{z}U\;=\;\;\text{MAC}\;\;kc, \end{array}$$

[9] $$\begin{array}{c} (d_{\mathrm{zz}}-k^{2})c\;=\;-k\psi , \end{array}$$

[10] $$\begin{array}{c} \left(d_{\mathrm{zz}}+d_{z}-k^{2}\right)b\;=\;-exp\left(-z\right)\;d_{z}U. \end{array}$$

Eq. **7** derives from the y-component of the Navier–Stokes Eq. **1** and shows that in regions where the electrical conductivity is sufficient the meridional circulation is controlled by electromagnetic forces resulting from the induced toroidal field (assuming that here no other significant force is acting on it). Note that in our model, we do not consider the region where the zonal flows are driven, which could be predominantly in the top atmospheric layers of the planet (29). Here, the Coriolis force of the meridional circulation balances the driving Reynolds stresses (or eddy momentum flux convergence). Eq. **7** holds in this form only where Reynolds stresses are absent. The electromagnetic force in Eq. **7** can be considered to be the antagonist to the Reynolds stresses; its role is to truncate the meridional flow at depth. It has been suggested that the breaking of inertia-gravity waves that are generated in the weather layer can serve as the antagonist (30), but it is not clear why the downward propagating waves should break.

Eq. **8** describes a thermal wind balance and derives from the y-component of the curl of Eq. **1**. Eq. **9** represents the balance of advection of codensity by the meridional flow and diffusion. Eq. **10** describes the balance between the induction of toroidal magnetic field, by the shearing of the imposed poloidal field due to gradients in the zonal flow (Ω-effect), and magnetic diffusion.

For a fixed wavenumber k the equations contain only a single control parameter which we call the MAC-number to express that the system is influenced by Magnetic, Archimedian (=buoyancy), and Coriolis forces:

[11] $$\begin{array}{c} \;\text{MAC}={\left(\frac{N}{\Omega }\right)}^{2}\frac{\sigma_{o}B^{2}d_{\sigma }^{2}}{2\rho_{o}\kappa }. \end{array}$$

We had found in 3D numerical simulations (27) that magnetic effects influence the decay of zonal winds in the stable layer by the parameter combination $\sigma B^{2}$. This concurs with this combination appearing in the MAC-number. In ref. 22, we found that, for fixed σ and B, a parameter $Ra_{\Omega }\propto N^{2}/\kappa$ controls the decay of the zonal flow when viscosity becomes insignificant. With the MAC-number we now combine all physical properties that influence the quenching of the zonal flow into a single parameter.

We assume that at z > 0 turbulent convection eradicates significant codensity differences, and we set c(z>0)=0. In ref. 22, we specified an ad hoc force in the y-direction, acting at z>0, for driving the zonal flow. Here, we simply set the wind velocity by a boundary condition U(z=0)=1. U=1 also holds for z>0, where c vanishes, according to Eq. **8**. For other applied boundary conditions and the technique for solving Eqs. **7**–**10**, see *Materials and Methods*.

### Results for the Simple Case.

**Fig. 2.**
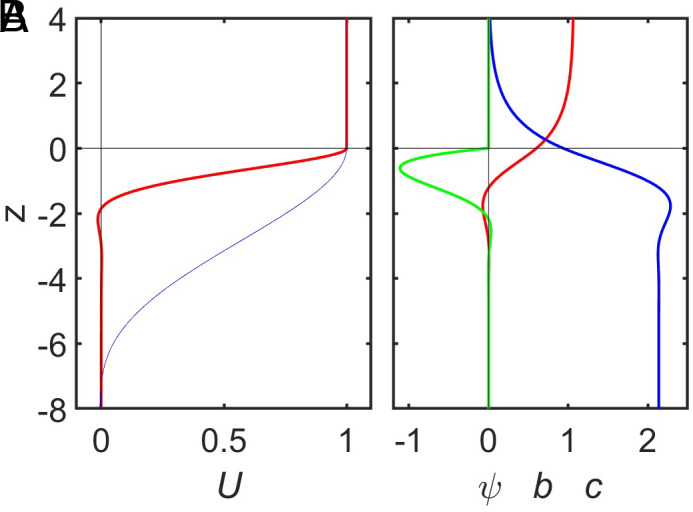
Solution for simple Boussinesq cases with exponential conductivity variation. Vertical coordinate z in units of conductivity scale heights. (*A*) Zonal flow amplitude U for MAC = 500 (red line) and MAC = 0.1 (thin blue line). (*B*) For MAC = 500 stream function ψ (red), toroidal magnetic field b (blue), codensity perturbation c multiplied by 1,000 (green).

We calculated results for MAC-numbers from 0.05 to 500,000. Values of order one are typical for 3D numerical simulations whereas in Jupiter MAC falls into the range 10^2^ to 10^5^ when the upper boundary of a stable layer is between 1,500 km and 3,000 km depth and N/Ω is of order one. We fix the horizontal wavenumber at k=π/20, which is a representative value for Jupiter. Fig. 2 shows depth profiles of the solution for MAC = 500. The wind velocity (Fig. 2*A*) drops strongly within a few conductivity scale heights below the stable layer boundary. Within a limited depth, interval there is a weak reverse flow. Deeper than six scale heights below the boundary the velocity is less than $10^{-4}$. The depth range over which the velocity drops depends on the MAC-number. The thin blue line in Fig. 2*A* represents the case for MAC = 0.1. The overshoot of the zero-line is more pronounced for the higher MAC-number, but otherwise, the general shape of the drop-off is similar. Also the meridional flow and the codensity perturbation drop to very small values at a few scale heights below the stable layer boundary (Fig. 2*B*). However, the toroidal magnetic field, which is induced by the velocity shear in the upper part of the stable layer, stays constant at greater depth in the region of rapidly increasing conductivity. Because for constant b(z) the LHS of Eq. **10** is constant, U(z) must decrease exponentially here at the same rate as the conductivity increases. To a limited degree, the toroidal field diffuses upward where it causes the meridional (vertical) flow to drop somewhat before it reaches the stable layer boundary.

**Fig. 3.**
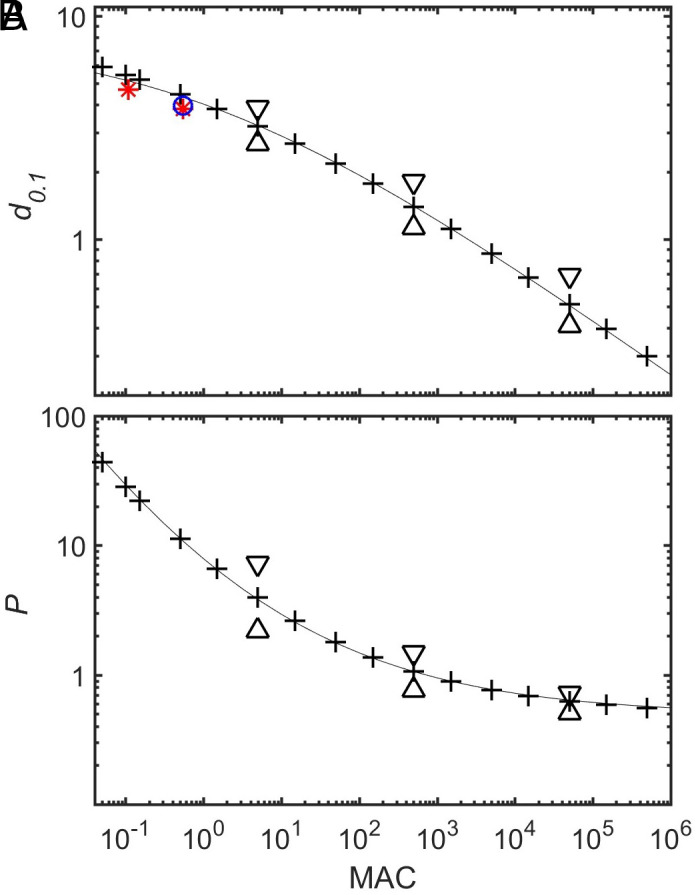
(*A*) Decay scale $d_{0.1}$ as a function of MAC-number for the simple Boussinesq case. Red stars show the results for 3-D simulations with N/Ω= 4.9 at Ekman-numbers of $10^{-5}$ and $2\times 10^{-6}$ and the blue circle with N/Ω= 10.9 and $E=10^{-5}$, see *Comparison with 3D Convection Simulations*. The line is the fit by Eq. **12**. (*B*) Power vs. MAC-number. The line is the fit by Eq. **14**. In addition to the standard wavenumber (crosses), for MAC = 500 also the results for k=π/10 (up-triangle) and k=π/40 (down-triangle) are shown.

To characterize the length scale of quenching of the zonal flow in the stable layer we determined the range over which U has dropped by a factor ten and call this the decay scale $d_{0.1}$. It decreases from about six conductivity scale heights at MAC = 0.05 to 0.3 scale heights at MAC = 500,000 (Fig. 3*A*), although not by a simple power-law. A good empirical fit, with a mean relative error of 2.4% for MAC≥1 is obtained by

[12] $$\begin{array}{c} d_{0.1}\;=\;\frac{8.09}{1+{\;\text{MAC}}^{1/4}}. \end{array}$$

Changing the horizontal wavenumber k has only a moderate influence on $d_{0.1}$ (triangles in Fig. 3).

The power needed for driving the jets must stay within reasonable limits. The total power balances the dissipation which is purely ohmic in our model, plus the work done by the meridional flow against buoyancy forces (see *SI Appendix* for their calculation). Fig. 3*B* shows the variation of driving power per unit surface area, scaled via

[13] $$\begin{array}{c} P=\tilde{P}\;\sigma_{o}B_{o}^{2}\kappa^{2}/d_{\sigma }, \end{array}$$

as function of the MAC-number. The nondimensional power drops with MAC, but varies only little at MAC-numbers larger than 1,000, leveling off toward a constant value near 0.5. A good fit with 2.5% mean relative error is given by

[14] $$\begin{array}{c} P\;=\;0.489\frac{{\;\text{MAC}}^{1/4}}{1+{\;\text{MAC}}^{1/4}}. \end{array}$$

### Comparison with 3D Convection Simulations.

We test the prediction that the quenching of zonal flows in a stable layer is governed by the parameter combination in the MAC-number with 3-D convection models for a rotating spherical shell. We briefly summarize the basic concept described in ref. 27. We consider a shell with ratio of inner to outer radius $r_{i}/r_{o}=0.7$. The region above $0.83r_{o}$ is convectively unstable, whereas at $r<0.83r_{o}$ the fluid is stably stratified. Full stability is reached below a transition region at $0.804r_{o}$. The conductivity varies exponentially with radius by a factor $10^{8}$. An axial dipolar magnetic field is imposed by a boundary condition at $r_{i}$. We assume fourfold symmetry in longitude, i.e., we simulate only one quarter of the full sphere, which has little influence on the character of the zonal jets and saves computing time. The relevant MHD-equations in the Boussinesq form (27) contain four basic control parameters, a Rayleigh number Ra, Ekman number E, Prandtl number Pr and magnetic Prandtl number Pm (see *Materials and Methods* for definitions).

In ref. 27, we kept the Ekman number at $10^{-5}$ and the degree of stability in the lower layer was set such that N/Ω= 4.9. Here, we add two more cases. We lower the Ekman number to $2\times 10^{-6}$ to weaken the influence of viscosity and to increase MAC by a factor of five. The same value of the MAC-number is obtained by setting N/Ω=10.9, $E=10^{-5}$. We keep the Prandtl numbers at Pr=Pm=0.5. The strength of the imposed field is set such that the local Elsasser number $\sigma_{i}B^{2}\left(r_{i}\right)/\left(\rho_{i}\Omega \right)$, evaluated at the inner boundary and at the poles, is 0.25. The Rayleigh number is chosen such that in each of the simulations the convective Rossby number $Ro_{c}=E{\left(Ra/Pr\right)}^{1/2}$ is kept at a value of 0.346. Given that we model strongly supercritical convection in the rapidly rotating regime, this ensures that the vigor of convection is comparable in the different cases.

**Fig. 4.**
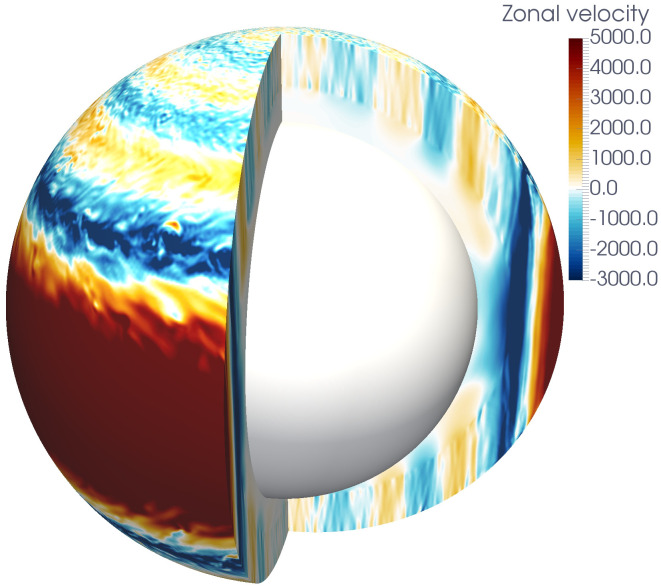
Snapshot of a 3D-simulation showing the azimuthal velocity component.

Fig. 4 illustrates the flow pattern for the case with $E=10^{-5}$. At the surface, it is dominated by bands of zonal wind that extend parallel to the rotation axis throughout the outer convection shell. Inside the tangent cylinder, associated with the boundary of the stable layer, the wind velocity drops rapidly with depth in the lower stable region.

To compare the results of the 3D convection models with the predictions of our simplified theory we calculate the decay scale $d_{0.1}$ as follows. As in ref. 27, we average the azimuthal velocities in the center of all jets inside the tangent cylinder, normalized with the velocity at the surface, to obtain a mean radial profile ${\overset{¯}{u}}_{\phi }$. The relevant value of the MAC-number expressed in terms of the parameters of the 3D-simulations is obtained as

[15] $$\begin{array}{c} \;\text{MAC}={\left(\frac{N}{\Omega }\right)}^{2}\sigma \left(r_{s}\right)B^{2}\left(r_{s}\right)\frac{\mathrm{Pr}}{2E}d_{\sigma }^{2}, \end{array}$$

where $r_{s}$ is the effective boundary of the stable layer. In the simple model this boundary is sharp, whereas in the 3D-models it is spread over a transition region. We choose $r_{s}=0.82r_{o}$, where the stable codensity gradient has reached 36% of its saturated value. The values of the MAC-number in the 3D-models are of order one. From the averaged profiles ${\overset{¯}{u}}_{\phi }$ we read the decay scale $d_{0.1}$ as the distance in radius over which the velocity has dropped to 10% of its value at $r_{s}$. The results, normalized with $d_{\sigma }$, are shown by the stars for the cases with N/Ω=4.9 and by the circle for N/Ω=10.9 in Fig. 3*A*. They agree well with the predictions from the simple inviscid and linearized Cartesian models. Although in the 3D models viscosity still plays a significant role, it hardly affects the decay scale of the winds, whereas the additional source of dissipation reduces their amplitude (*SI Appendix*).

## Models Adapted to Jupiter

In order to make the linearized Cartesian model directly applicable to gas planets we extend it in two respects. We now study the compressible case instead of the Boussinesq limit, and we allow for arbitrary functions for the reference density $\tilde{\rho }\left(z\right)$ and electrical conductivity σ(z). Furthermore, we allow for an angle θ between rotation vector and the z-direction (the direction of gravity) and angle γ between magnetic field and z, to account for conditions at different latitudes (Fig. 1). The resulting equations are given in *Materials and Methods*.

We model the outer 5,600 km of Jupiter from the 1-bar level down to 0.92$r_{J}$ ($r_{J}\approx$ 70,000 km is Jupiter’s mean radius), and study different radii $r_{s}$ for the upper boundary of the stable layer. We employ analytical approximations for the electrical conductivity data in refs. 8 and 22 and for the density model of (31) (*SI Appendix*). For the characteristic latitudinal wavelength of the zonal jets inside the tangent cylinder, we pick 10,000 km. The angle γ between magnetic field and the vertical at colatitude θ is calculated for a polar dipole via cotγ=−2tanθ. The field strength is obtained as $B=g_{1}^{0}\;{\left(r_{J}/r_{s}\right)}^{3}\;{\left(4\;\;{\text{cos}}^{2}\theta +\;{\text{sin}}^{2}\theta \right)}^{1/2}$, with the Gauss coefficient $g_{1}^{0}=0.41$ mT. Jupiter’s rotation frequency is $\Omega =1.75\times 10^{-4}s^{-1}$. We fix the degree of stability to N/Ω=1 and set $\kappa =1.5\times 10^{-3}m^{2}/s$ (22). For the velocity amplitude of the jets inside the tangent cylinder we take $U_{o}$= 25 m/s, although closer to the equator it is larger. As the jet velocity is set to a nondimensional value of one in the calculations, the scaling factors in Eqs. **5** and **6** must be multiplied with $U_{o}d_{\sigma }/\kappa$ to convert the results into physical units. The power varies quadratically with $U_{o}$, hence its scaling factor (Eq. **13**) is multiplied by ${\left(U_{o}d_{\sigma }/\kappa \right)}^{2}$. Note that in the modified scaling factors the (effective) diffusivity κ drops out.

**Fig. 5.**
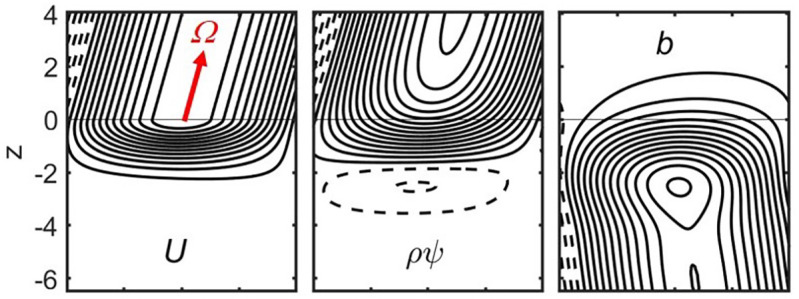
Solution for a Jupiter-like case with the stable layer boundary at 0.975$r_{J}$ for $\theta =45^{{}^{\circ}}$, $\gamma =-26.6^{{}^{\circ}}$. Isocontours of wind velocity U (*Left*), meridional mass flux $\tilde{\rho }\psi$ (*Middle*), toroidal magnetic field b (*Right*). Note that the current associated with the toroidal field follows the contour lines of b. The vertical coordinate is exaggerated threefold compared to the horizontal one. The z-coordinate is in multiples of the conductivity scale height at the boundary, 158 km for 0.975$r_{J}$.

Fig. 5 illustrates some properties for the case with $\theta =45^{{}^{\circ}}$ and $r_{s}=0.975r_{J}$ in the vicinity of the stable layer boundary. The MAC-number calculated with local properties (σ, B, $d_{\sigma }$, and $\tilde{\rho }$) at $r_{s}$ is 147. Above $r_{s}$ (or z=0) the wind velocity and also the meridional flow are aligned with the direction of rotation. U drops sharply within two conductivity scale heights below $r_{s}$. The induced toroidal magnetic is aligned with the direction of the imposed poloidal field at greater depth. In terms of absolute numbers, the meridional flow is weaker than the zonal flow by at least five orders of magnitude and the toroidal field is weaker than the poloidal field by one order of magnitude.

**Fig. 6.**
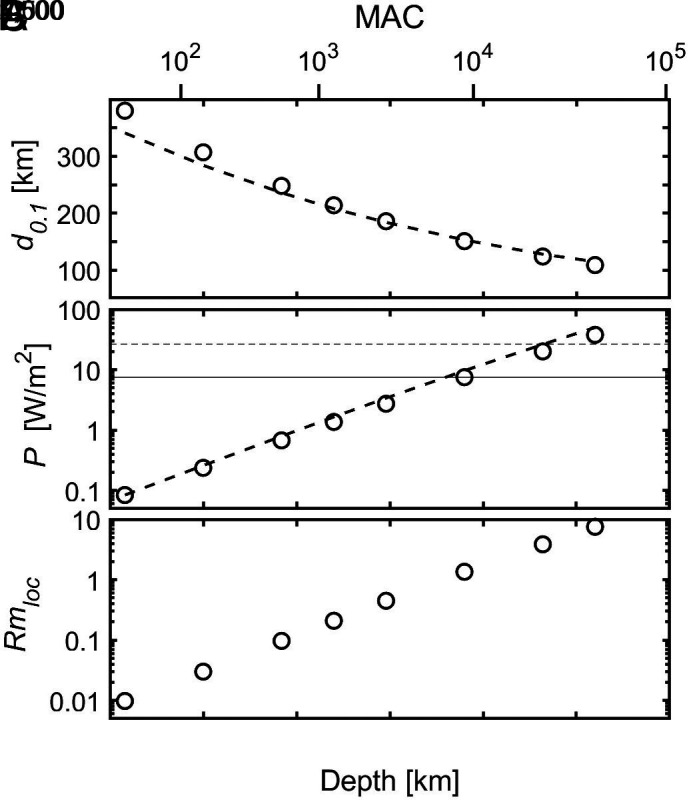
Properties of the Jupiter-like case at mid-latitude as function of the depth of the stable layer boundary for a wind velocity of 25 m/s. (*A*) Decay range $d_{0.1}$, (*B*) driving power, (*C*) maximum value of the local magnetic Reynolds number. The broken lines in (*A*) and (*B*) are the fits as function of MAC to the simple Boussinesq cases by Eqs. **12** and **14**, respectively. The thin full line in (*B*) is the observed internal heat flow and the broken line is the upper bound for the total dissipative heating according to ref. 32. The MAC-number for different depths of the boundary is shown on the *Top* panel.

For jets at mid-latitude ($\theta =45^{{}^{\circ}}$) several properties of interest have been calculated for different depths of the stable layer boundary in the range from 1,540 km to 2,800 km. The electrical conductivity $\sigma_{o}$ rises from $10^{-3}$ S/m to 16.7 S/m between 1,540 km and 2,800 km depth; the corresponding scale heights $d_{\sigma }$ are 145 km and 280 km, respectively. The distance $d_{0.1}$ over which the jet velocity drops to 10% of its surface value decreases from 400 km to 100 km (Fig. 6*A*). The power per unit area for driving a jet with 25 m/s velocity amplitude increases steeply with the depth of the stable layer boundary (Fig. 6*B*), as was found before (22). For a boundary shallower than 2,000 km, it is less than 1 W/m^2^, whereas for a depth greater than 2,400 km it exceeds the mean interior heat flow from Jupiter of 7.5 W/m^2^ (33). The broken lines in Fig. 6 *A* and *B* show the values calculated from Eqs. **12** and **14**. Even though they are derived from a simpler Boussinesq model with Ω‖B‖g, they fit the results of the Jupiter-like models well. Hence the MAC-number controls the essential physics of the quenching of winds in a weakly conducting stable layer also in the more realistic case. It suggests that Eqs. **12** and **14** can serve as a guide to assess the influence of changes in any of the physical parameters that play a role in the process.

**Fig. 7.**
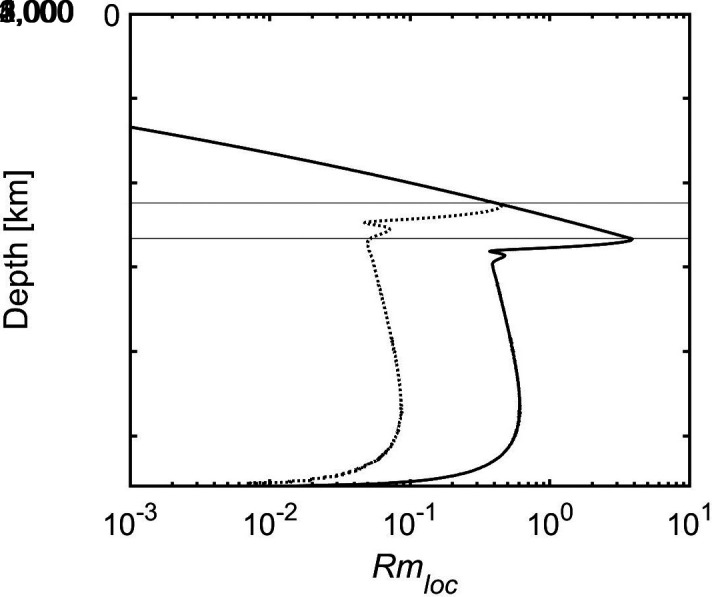
Local magnetic Reynolds number as function of depth for two Jupiter-like cases at mid-latitude with surface velocity amplitude U= 25 m/s and stable layer boundary at 2,660 km (full line) and 2,240 km depth (dotted).

The poloidal magnetic field will be modified by the zonal flow when the magnetic Reynolds number calculated with local values, $\;{\text{Rm}}_{\;\text{loc}}=\mu_{o}\sigma d_{\sigma }U$, reaches or exceeds order one at some depth. Fig. 7 shows how $\;{\text{Rm}}_{\;\text{loc}}$ varies with depth for two cases. It peaks right below the stable layer boundary and remains at roughly one tenth of the peak value throughout the stable layer. $\;{\text{Rm}}_{\;\text{loc}}$ rises strongly with increasing depth of the stable layer boundary in pace with the associated increase of electrical conductivity (Fig. 6*C*). Its peak value exceeds one when the boundary is deeper than 2,400 km.

**Fig. 8.**
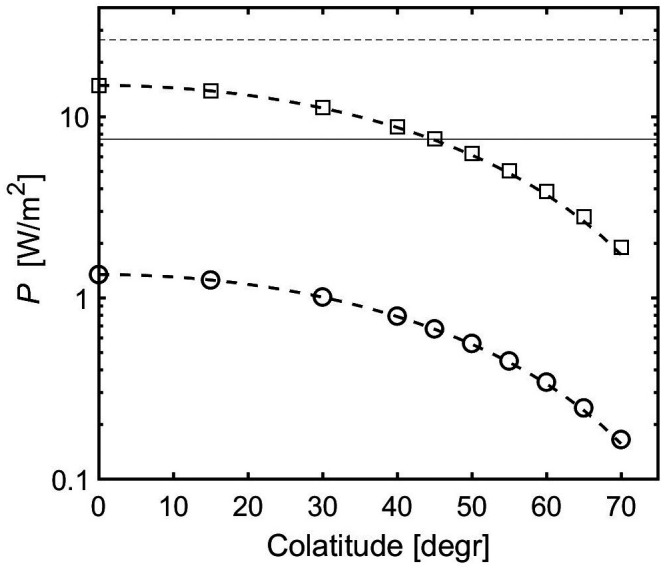
Power as function of colatitude for two Jupiter-like cases with surface velocity amplitude U= 25 m/s and stable layer boundary at 1,960 km (circles) and 2,450 km depth (squares). The broken lines represent a variation with ${\text{cos}}^{2}\theta$, anchored at the value for θ= 0. Thin horizontal lines as in Fig. 6*B*.

We studied the dependence of the solution on latitude in the range from $\theta =0^{{}^{\circ}}$ to $70^{{}^{\circ}}$, the latter value being close to the edge of the tangent cylinder, but still within. The decay scale $d_{0.1}$ is almost insensitive to a change of latitude. As a consequence, also the peak value of the local magnetic Reynolds number is unaffected. A strong effect is found for the power requirement of the zonal flow, which becomes smaller at lower latitudes (Fig. 8). The power P varies almost as ${\text{cos}}^{2}\theta$, as shown by the broken lines. The amplitude of the meridional flow ψ and of the induced toroidal field b also vary with colatitude, approximately as cos θ. Their covariation with P is expected, since the meridional flow amplitude is controlled by the driving Reynolds stresses, and ψ and b are directly linked by Eqs. **7** and **16**.

The strong dependence of P on latitude can partly be traced back to the variation of the dipole field strength with θ, because the scaling factor for power (Eq. **13**) contains $B^{2}$. However, from the pole to $\theta =70^{{}^{\circ}}$ the power is found to drop by a factor of eight, whereas $B^{2}$ varies only by a factor of three. Therefore, the change of orientation between gravity, rotation, and magnetic field vectors also has an influence on the dissipation. Simple (Boussinesq) test cases in which θ and γ are varied independently show that the angle between gravity and the magnetic field is key. Setting $\theta =0^{{}^{\circ}}$, $\gamma =90^{{}^{\circ}}$ results in a reduction of power by a factor of eight compared to $\theta =\gamma =0^{{}^{\circ}}$. Increasing θ while keeping $\gamma =0^{{}^{\circ}}$ has only a minor effect.

The vertical (meridional) velocity is only a small fraction of a mm/s in the stable layer. The meridional mass flux, or the product $\tilde{\rho }\psi$, stays constant with z according to Eq. **16** at shallow depth where b is essentially zero. Because of the sharp drop of density with radius in the outer regions of the planet, the velocity increases toward the surface. The constancy of mass flux holds as long as the driving force for the zonal winds, i.e., the Reynolds stress, remains negligible. If we assume that the winds are mostly driven by vigorous moist convection near the top of the atmosphere, down to a pressure level of 6 bars (57 km depth) (29), we can calculate the vertical velocity at this depth assuming constant $\tilde{\rho }\psi$ below. For a stable layer boundary at 2,100 km, it is 0.5 mm/s. This gets larger (smaller) by an order of magnitude for 2,550 km (1,650 km) boundary depth. These estimates for the vertical velocity are an upper limit if Reynolds stresses in deeper parts of the convection zone contribute substantially to the driving of the jets.

## Discussion and Conclusions

The MAC-number proves to be the primary control parameter for the quenching of zonal winds and the power required to drive them. We derived its influence for a simple setting, i.e., using linearized equations in the Boussinesq approximation with aligned gravity, rotation axis, and magnetic field. A comparison of the predictions of this simple model with the results of 3-D simulations of convection-driven jets, and with linearized simulations for a density-stratified fluid with different angles between the relevant vectors, shows that the MAC-number is indeed the essential parameter also in less idealized cases. We can use it to assess the consequences of variations in properties that are not well known. Double-diffusive convection in the stable layer, probably in the form of stacked sublayers, is needed to transport the internal heat of the planet without excessive temperature gradients (34). This can be parameterized by an effective diffusivity κ, for which we adopted the value estimated in ref. 22. However, this value is merely an educated guess. An increase or decrease of κ by an order of magnitude alters MAC by a factor of 10. In the relevant range for the MAC-number this implies a change of the wind decay scale and of the driving power by a factor of 1.7 at most. The same holds for variations in the stabilizing density gradient. Therefore the result is not strongly sensitive to the adopted values of these poorly constrained parameters. However, for a weakly stable stratification with N/Ω<1 the shear flow in the stable layer may be subject to Kelvin–Helmholtz instabilities (*SI Appendix*).

The characteristic drop-off length scale for the winds depends moderately on the depth of the stable layer boundary. It lies in the range of 100 to 300 km (Fig. 6*A*), i.e., it is rather sharp and substantially smaller than the depth of the overlying region where the wind velocity is constant. In the best-fitting solutions to the observed gravity anomalies the drop-off scale is typically larger than this (e.g., extended data figure 2 in ref. 4), although when additional magnetic constraints are included it becomes sharper. The discontinuous onset of stability in our models favors a rather sharp drop-off. In the case of a gradual increase of stability with depth the decay of the jet velocity can be expected to be more smeared out.

The power required for driving the wind and the peak value of the magnetic Reynolds number associated with the winds depend strongly on the location of the stable layer. This allows us to put constraints on the depth extent of the winds in addition to those based on the gravity signal. For the dissipation (power) the internal heat flow from Jupiter is frequently considered to be an upper limit. On thermodynamic grounds, the total dissipation could be up to 3.6 times larger than the internal heat flow (32). This comprises also the dissipation associated with turbulent convection and with the dynamo process at greater depth. Therefore it appears unlikely that the dissipation linked to the zonal winds substantially exceeds the internal heat flow. The latter would be the case if the stable layer boundary was deeper than 2,500 km (Fig. 6*B*). This limit could be shifted somewhat, but not more than a few hundred kilometers, if the effective diffusivity or the degree of stability differed by an order of magnitude from the adopted values. Rather strong distortions of the nonaxisymmetric components of Jupiter’s magnetic field by the winds must be expected if the local magnetic Reynolds number of the wind significantly exceeded one at some depth (6). Such distortions are not observed. Taking $\;{\text{Rm}}_{\;\text{loc}}=3$ as an upper limit requires the stable layer boundary to be no deeper than 2,600 km, which is almost identical to the constraint based on the driving power.

A shallow boundary is not in conflict with constraints from the dissipation or magnetic field properties. Explaining the observed gravity signal with a truncation depth at 1,500 km would require wind velocities that are twice larger than those observed at the cloud level (3). A similar increase of velocity from the surface to 50 km depth has been found by the Galileo probe at low latitudes (35). Should this be the case also at higher latitudes, a stable layer reaching up to 1,500 km would be possible. The dissipation associated with the winds would then be only a small fraction of the internal heat flow.

The flux of energy Q from small-scale eddies into the mean flow, per unit mass of the fluid involved, has been calculated from the observed eddy momentum flux $\rho \langle u_{x}^{\prime }u_{y}^{\prime }\rangle$ at the cloud level (10, 36), where $u^{\prime }$ denotes the horizontal components of the eddy flow and the angular bracket stands for the spatial average. Here, we adopt the range of $Q\;=\;\left(7-12\right)\;\times \;10^{-5}$ W/kg (10). A similar value of has been derived with a diagnostic based on potential vorticity (37). To arrive at the power driving the zonal flow an estimate of the mass involved in the eddy motion is needed. If $\left\langle u_{x}^{\prime }u_{y}^{\prime }\right\rangle$ stays at the surface value, from a reference pressure $p_{o}$ down to a pressure level p, and drops to zero below, the total power is given by $P=Q(p-p_{o})/g$, where g = 25 m/s^2^ is Jupiter’s gravity. Setting $p_{o}$= 1 bar, the pressure at the bottom of the region in which the jets are driven would be at 1.3 bar, 4 bar, and 31 bar for a power of 0.1, 1, and 10 W/m^2^, respectively, corresponding to depths of 7 km, 42 km, and 150 km. The first value is substantially smaller than the thickness of the weather layer. It seems unlikely that deeper parts of the zone of moist convection do not contribute to the eddy momentum flux, which makes a very shallow stable layer boundary, requiring little power, less likely. The middle value fits roughly the zone of moist convection. For the highest value, the zone of intense driving of the mean flow had to extend deeper than the weather layer. In addition to the other arguments this disfavors a deep stable layer boundary, provided that Reynolds stresses associated with convection below the weather layer are not important. With this reservation, the most probable depth to the stable region and the point where the zonal flows are quenched is therefore around 2,000 km.

All previous conclusions regarding the depth of the zonal winds depend on the applicability of the conductivity profile that we use (8). A more general conclusion would be that the stable layer boundary is likely located at the depth where the electrical conductivity reaches approximately 0.01 to 0.03 S/m (typical conductivity of freshwater). For example, with the conductivity model of ref. 38, the preferred depth would shift from 2,000 km to 2,400 km.

A deep meridional flow is an essential part of our model. The presence of meridional flow in Jupiter, down to a level of at least 240 bars and organized in a set of Ferrel-like cells associated with the zonal winds, has been inferred from anomalies in the ammonia concentration observed by the microwave instrument on Juno (23). These anomalies have been attributed to vertical advection of ammonia concentration gradients. Our models with a stable layer boundary near 2,000 km predict a radial velocity of the meridional flow at the 6-bar level of 0.5 mm/s. An interesting question is whether such slow flow is sufficient to cause the observed ammonia anomalies.

The significant decrease with colatitude of the dissipation associated with a jet of given velocity (Fig. 8) can be part of the reason why the low-latitude jets inside the tangent cylinder are faster than those at mid- to high-latitudes. This assumes that there is little transfer of energy between different latitudinal bands, which seems likely because the winds are driven by a local eddy momentum flux and extend along geostrophic cylinders into the region where their energy is dissipated. However, reduced dissipation is unlikely to be the sole reason for faster jets at low latitudes. The analysis from cloud-tracking shows a good correlation of the eddy momentum flux with the velocity of the jets (10). Furthermore, in the concept of zonostrophic turbulence (e.g., ref. 39) the rate of transfer depends on the parameter β∝2Ωsinθ, which decreases with latitude. Still, the latitudinal variation of dissipation may contribute to the vigor of the jets near $\pm 20^{{}^{\circ}}$ latitude.

The existence of a stable layer at the required depth in Jupiter is hypothetical. However, we are not aware of a suitable mechanism without the stable layer for quenching the zonal winds at a depth that is consistent with the gravity and magnetic field data, and that is compatible with reasonable limits on the driving power. Direct quenching of the zonal flows by electromagnetic forces in the absence of stable stratification was found to entirely suppress jets inside the tangent cylinder (14, 15, 22). In order to consolidate or falsify our model, independent evidence on the existence, depth, and degree of stratification of such layer would be highly desirable. Such evidence might come from observations of normal modes of Jupiter (40). The framework that we developed here can also be applied to other objects with similar zonal surface winds, such as Saturn and possibly gaseous exoplanets.

## Materials and Methods

### Anelastic Model for Arbitrary Angles Been z, Ω and B.

Using complex notation for all variables and allowing for arbitrary functions σ(z) and $\tilde{\rho }\left(z\right)$ Eqs. **7**–**10** can be generalized, where now the meridional velocity relates to the stream function by $\tilde{\rho }u=\nabla \times \left(\tilde{\rho }\psi e_{y}\right)$:

[16] $$\begin{array}{c} 2\left[\cos\theta \;d_{z}+\sin\theta \;ik\right]\left(\tilde{\rho }\psi \right)\;=\;\left(\cos\gamma \;d_{z}+ik\sin\gamma \right)b, \end{array}$$

[17] $$\begin{array}{c} (\cos\theta \;d_{z}+\sin\theta \;ik)U\;=\;\;\text{MAC}\;ikc, \end{array}$$

[18] $$\begin{array}{c} (d_{\mathrm{zz}}+d_{\rho }^{-1}d_{z}-k^{2})c\;=\;ik\psi , \end{array}$$

[19] $$\begin{array}{c} \left(d_{\mathrm{zz}}-d_{\sigma }^{-1}d_{z}-k^{2}\right)b=-\sigma \left(\cos\gamma \;d_{z}+\sin\gamma \;ik\right)U. \end{array}$$

Note that the scale height $d_{\sigma }$ is negative as defined here. The MAC-number (Eq. **11**) is calculated with the reference values of B, $\tilde{\rho }$, σ, and $d_{\sigma }$ taken at the upper boundary of the stable layer.

### Boundary Conditions.

For the Jupiter-like model we map the region $0.92r_{J}<r<r_{J}$ onto the nondimensional range $z_{l}<z<z_{u}$ with z=0 at the stable layer boundary. In the simple Boussinesq models, we picked $|z_{l}|$ and $z_{u}$ large enough so that their choice did not affect the solution. We deal with a sixth-order boundary value problem and must specify six boundary conditions. These are U(0)=1, c(0)=0, $U\left(z_{l}\right)=d_{z}\psi \left(z_{l}\right)=c\left(z_{l}\right)=0$, and $b(z_{u})=0$.

### Solving the Linearized Equations.

The boundary value problem is stiff, in particular for large values of the MAC-number and/or steep variation of conductivity and density covering many orders of magnitude. It requires special measures for a stable solution. We employ quad-accuracy for floating point numbers and a multiple shooting method (41) based on fourth-order Runge–Kutta integration with up to ten internal matching points in the stable layer.

### 3D Simulations.

The Rayleigh number, Ekman number, Prandtl number, and magnetic Prandtl number are defined as

[20] $$\begin{array}{c} Ra=\frac{\alpha gd^{4}d\tilde{C}/dr}{\kappa \nu }\;\;\;E=\frac{\nu }{\Omega d^{2}}\;\;\;\;Pr=\nu /\kappa \;\;\;\;Pm=\mu_{o}\sigma_{o}\nu , \end{array}$$

where α is thermal expansivity, $d\tilde{C}/dr$ the imposed background codensity gradient in the unstable layer, d the shell thickness, and $\sigma_{o}$ the conductivity at $r_{i}$. Simulations were conducted with the code MAGIC (https://magic-sph.github.io/). See ref. 27 for details of the simulations.

## Supplementary Material

Appendix 01 (PDF)

## Data Availability

All 3D simulations were carried out using the magnetohydrodynamic code MagIC which is open source and available in Zenodo (42). This study used the adapted version (43) from (27).
