# Supplementary material for: Quenching of zonal winds in Jupiter’s interior
Source: Proc Natl Acad Sci U S A. 2024 Jun 10;121(25):e2402859121. doi: 10.1073/pnas.2402859121 (PMC11194569; doi:10.1073/pnas.2402859121)
Supplement: Supplementary file 1 — Appendix 01 (PDF) [file pnas.2402859121.sapp.pdf]

Supplementary Information for

Quenching of zonal winds in Jupiter’s interior

Ulrich R. Christensen and Paula N. Wulff

Ulrich R. Christensen.
E-mail: christensen@mps.mpg.de

This PDF file includes:

Supplementary text
Figs. S1 to S2
References for SI reference citations

Supporting Information Text

Influence of viscosity on the zonal winds in 3D models. We compare three different levels of model idealization, at Mac ≈ 0.1 in the Boussinesq limit. The most complex case is the full 3D-model with convective driving of the winds as described in the main text, at an Ekman number 10^{-5} and N/Ω=4.9. The simplest case is our inviscid model with aligned magnetic field, rotation and gravity. Intermediate is a cartesian model that includes viscous friction, calculated as described in (2). If we re-introduce viscosity, eqns. 7 and 8 of the main text are replaced by

$$2d_z\psi \;=\; -d_z b \;-\; \frac{E^*}{\Lambda^*}\,(d_{zz}-k^2)U \qquad [1]$$

$$d_zU \;=\; \text{Mac}\;kc - \frac{1}{2}E^*\Lambda^*(d_{zzzz}-2k^2d_{zz}+k^4)\psi \quad , \qquad [2]$$

where E^* and Λ^* are (local) values of the Ekman number and Elsasser number, respectively, based on the conductivity scale height and in case of the Elsasser number the magnetic field strength and conductivity at the stable layer boundary. In the 3-D simulation their values are $E^* = 3.4 \times 10^{-3}$ and $\Lambda^* = 6.1 \times 10^{-5}$. Both are small compared to one, which suggests that the viscous effects in eqn. 2 are insignificant. The ratio which appears in eqn. 1, which is equivalent to an inverse Chandrasekhar number, is 56, showing that at these parameters viscosity plays a significant role for shaping the meridional flow in the stable layer. For our viscous cartesian model we picked this value of E^*/Λ^*.

Figure S1 compares the variation of zonal flow with depth for the three models. In the 3D-case the normalized mean profile of the jets at mid-to-high latitudes, inside the tangent cylinder, calculated as described in (3), is displayed. The profiles for the inviscid and the viscous cartesian models show only minute differences, even though the viscous dissipation is slightly larger than the ohmic dissipation in the case that includes viscosity. The shape of the velocity decrease in the 3-D model also agrees surprisingly well given the various differences to the cartesian models. A reason for the steeper decay near the top of the stable layer in the 3-D case could be that in the cartesian models the codensity anomaly at $z = 0$ is set to zero, whereas in the 3-D models this is not the case.

Our comparison strenghtens the finding that convectively driven 3-D models, which are necessarily far more viscous than is realistic for planets, still correctly capture the decay length scale of the zonal wind in a weakly conducting stable layer. Of course, the additional dissipation will, for the same level of driving Reynolds stresses, reduce the zonal wind velocity. Qualitatively this effect can be seen in Fig. S2, showing profiles of the surface zonal wind as function of latitude for the three 3-D models that we discuss. Velocity is here expressed as a Rossby number, i.e., scaled by Ωd. When lowering the Ekman number from 10^{-5} to 2×10^{-6}, hence reducing the influence of viscous friction, the amplitude of the jets at mid and high latitudes increases by at least 50%. When instead the value of N/Ω is increased from 4.9 to 10.9, which results in the same value of the Mac number as in the case with lower Ekman number, the amplitude drops slightly. In this case the increased value of Mac results in a steeper gradient of the zonal velocity, which implies, for an unchanged viscosity value, more viscous friction.

Stability of vertical zonal wind shear. The large amplitudes and the rather steep decrease of the zonal wind velocity in the stable layer begs the question if the flow could be subject to Kelvin-Helmholtz instabilities. The requirement for the shear flow being stable is that the Brunt-Väisälä frequency N is larger than $1/2\,dU/dz$ (4). Approximating $dU/dz \approx U_o/d_{0.1}$ we find that for our nominal values of U_o= 25 m/s and $N/\Omega = 1$ the decay length scale $d_{0.1}$ must be larger than 70 km for stability, which is fulfilled for all reasonable values for the depth of the stable layer boundary. However, the speed of the northern off-equatorial jet exceeds 100 m/s, requiring $d_{0.1} > 300\ km$, which for $N/\Omega = 1$ and the electrical conductivity profile of (5) is only possible when the top of the stable layer is shallower than 1750 km (Fig. 6a). Assuming a larger degree of stability eases the situation, even though it implies a larger Mac number, hence a slightly smaller decay scale, when everything else is equal. With $N/\Omega = 2$ the upper boundary of the stable layer could be as deep as 2200 km to preserve stability. Conversely, a value of N/Ω significantly less than one seems unlikely if we assume that the northern off-equatorial jet is not Kelvin-Helmholtz unstable, which would certainly come with enhanced dissipation.

Methods.

Calculation of power. The total power needed to drive the zonal flow can be calculated from the ohmic dissipation and the work done against buoyancy by the meridional flow. The non-dimensional ohmic dissipation D, with the scaling given by eqn. 13 in the main text, is

$$D = \frac{1}{2}\int \sigma^{-1}(d_zb\;d_zb^* + k^2 b\;b^*)dz \quad , \qquad [3]$$

where the asterisk indicates the complex conjugate. The work done against buoyancy forces is

$$P_{buoy} = -Mac \int Re[ik\tilde{\rho}\psi c]\,dz \quad , \qquad [4]$$

where Re stands for the real part. The total dimensionless power is $P = D + P_{buoy}$. For small values of the Mac number both contributions are comparable, whereas for Mac ≥ 500, P_{buoy} contributes less than 20 %.

Parameterization of density. In the Jupiter-like cartesian models the density values of (1) are approximated by

$$\rho(r) = 3.19 \times 10^7 \frac{kg}{m^3}\ exp\left(\frac{-0.39438}{12.55438 - 12.5\, r/r_J} - 11.80\, \frac{r}{r_J}\right)\quad, \qquad [5]$$

which fits the data points within 10% relative deviation.

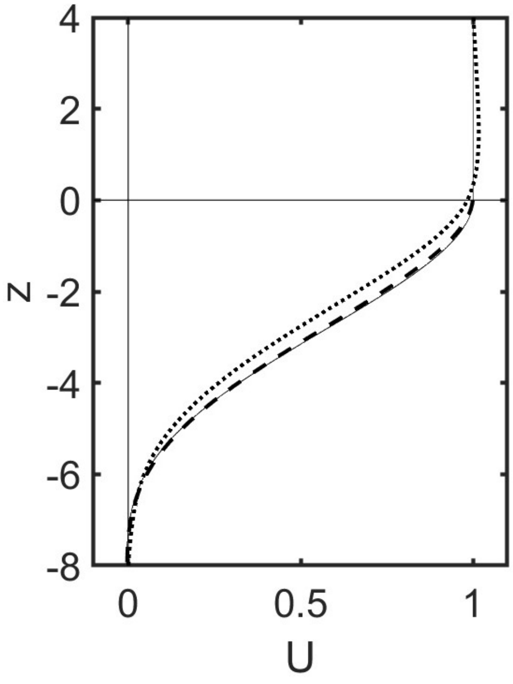

Fig. S1. Zonal wind velocity U as function of depth. The thin full line is for the inviscid cartesian model, the broken line for the cartesian model including viscosity, both at Mac=0.1. The dotted line is the mean profile inside the tangent cylinder for the 3-D simulation at E=10^{-5} and N/Ω=4.9 with a nominal Mac-number of 0.108. Depth is scaled with the conductivity scale height.

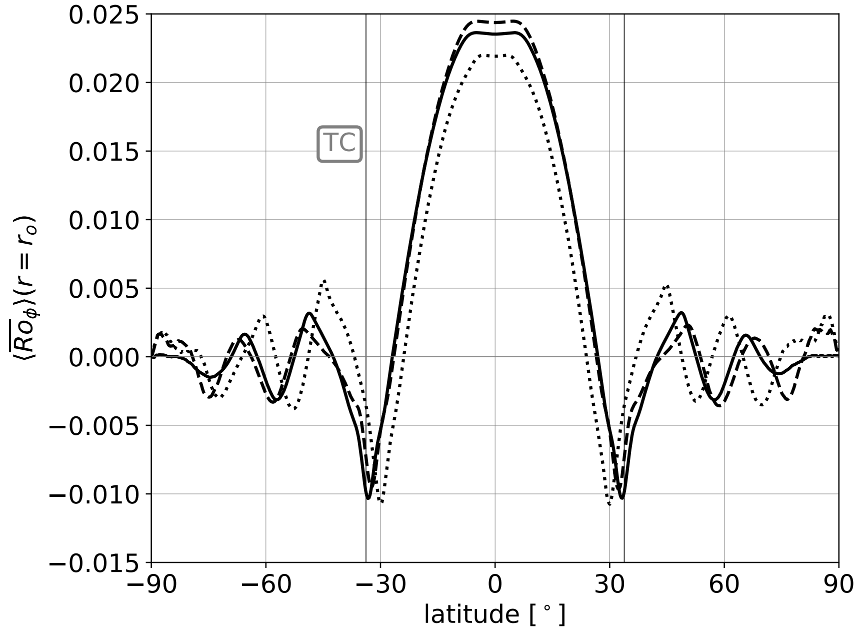

Fig. S2. Time-averaged zonal velocity, scaled by Ωd, at the surface of the 3D simulations versus latitude. Full line for E=10^{-5}, N/Ω=4.9, dotted line for E=2×10^{-6}, N/Ω=4.9, and broken line for E=10^{-5}, N/Ω=10.9.

References

1. N. Nettelmann, A. Becker, B. Holst, and R. Redmer. Jupiter models with improved ab-initio hydrogen equation of state H-REOS.2. *Astrophys. J.*, 750:52, 2012.
2. U. R. Christensen, J. Wicht, and W. Dietrich. Mechanisms for limiting the depth of zonal winds in the gas giant planets. *Astrophys. J.*, 890:61, 2020.
3. P. N. Wulff, U. R. Christensen, W. Dietrich, and J. Wicht. Effects of a stably stratified region with radially varying conductivity on the formation of zonal winds on gas planets. *J. Geophys. Res.*, 2024.
4. J. S. Turner. *Buoyancy effects in fluids*. Cambridge Univ Press, Cambridge, 1973.
5. M. French, A. Becker, W. Lorenzen, N. Nettelmann, M. Bethkenhagen, J. Wicht, and R. Redmer. Ab initio simulations for material properties along the Jupiter adiabat. *Astrophys. J. Suppl.*, 202:5, 2012.
